# Effect of Pretreatments on the Enzymatic Hydrolysis of High-Yield Bamboo Chemo-Mechanical Pulp by Changing the Surface Lignin Content

**DOI:** 10.3390/polym13050787

**Published:** 2021-03-04

**Authors:** Lianxin Luo, Xiaojun Yuan, Sheng Zhang, Xuchong Wang, Mingfu Li, Shuangfei Wang

**Affiliations:** 1 College of Light Industry and Food Engineering, Guangxi University, Nanning 530004, China; luolianxin@gxu.edu.cn (L.L.); 1816301034@st.gxu.edu.cn (X.Y.); 1916301042@st.gxu.edu.cn (S.Z.); 1916301029@st.gxu.edu.cn (X.W.); 2 Guangxi Key Laboratory of Clean Pulp & Papermaking and Pollution Control, Guangxi University, Nanning 530004, China; 3 University of Chinese Academy of Sciences, Beijing 100049, China; 4 Ningbo Institute of Materials Technology & Engineering, Chinese Academy of Sciences, Ningbo 315201, China

**Keywords:** bamboo, high-yield pulp, pretreatment, enzymatic hydrolysis, surface lignin content, methylene blue adsorption

## Abstract

Hydrogen peroxide chemo-mechanical pulp (APMP), sulfonated chemo-mechanical pulp (SCMP), and chemical thermomechanical pulp (CTMP) were used as raw materials to explore the effects of hydrogen peroxide (HP), Fenton pretreatment (FP), and ethanol pretreatment (EP) on the enzymatic hydrolysis of high-yield bamboo mechanical pulp (HBMP). The surface lignin distribution and contents of different HBMPs were determined using confocal laser scanning microscopy (CLSM) and X-ray photoelectron spectroscopy (XPS). The correlation between the surface lignin and the enzymatic hydrolysis of HBMP was also investigated. The residue of enzymatic hydrolysis was used to adsorb methylene blue (MB). The results showed that the cracks and fine fibers on the surface of APMP, SCMP, and CTMP increased after FP, when compared to HP and EP. The total removal content of hemicellulose and lignin in SCMP after FP was higher than with HP and EP. Compared to SCMP, the crystallinity increased by 15.4%, and the surface lignin content of Fenton-pretreated SCMP decreased by 11.7%. The enzymatic hydrolysis efficiency of HBMP after FP was higher than with HP and EP. The highest enzymatic hydrolysis of Fenton-pretreated SCMP was 49.5%, which was higher than the enzymatic hydrolysis of Fenton-pretreated APMP and CTMP. The removal rate of MB reached 94.7% after the adsorption of the enzymatic hydrolysis residue of SCMP. This work provides an effective approach for a high value-added utilization of high-yield bamboo pulp.

## 1. Introduction

Non-renewable fossil energy and fuels cannot meet the development of the current social productivity with the increase in the demand for energy. As an important part of renewable energy, biomass resources can be converted into power energy after decomposition, which is of great significance for alleviating the energy crisis. Hardwood and softwood raw materials with a high cellulose content and less impurities are ideal raw materials for enzymatic hydrolysis; however, the life cycle is too long, and these are used as building materials to meet people’s needs, and so it limits the wood raw materials to produce biofuels, such as bioethanol.

Bamboo, as a perennial grass, is found in East and Southeast Asia. In China, the bamboo forest is a type of economic forest, covering an area of 3.19 million hm^2^, which can produce 1356 billion bamboo shoots each year [1]. Compared with agricultural waste, such as wheat straw and straw, bamboo has higher cellulose and hemicellulose contents. Therefore, using bamboo as a raw material to produce fermentable sugar presents a great development prospect.

The production of bioethanol from lignocellulosic materials requires three key steps, including pretreatment, enzymatic hydrolysis, and fermentation. The key issue in the conversion of lignocellulose to bioethanol is the development of effective and economic pretreatment and enzymatic hydrolysis methods [2,3]. The purpose of pretreatment is to destroy the rigid crystal structure of the raw material and to remove or reduce the hemicellulose and lignin.

In recent years, a number of pretreatments have been developed. Acid pretreatment is a low-cost pretreatment method suitable for industrial production; however, the pseudo-lignin generated during acid treatment led to the non-productive adsorption of lignin on the enzyme, thus, greatly reducing the enzymatic hydrolysis [4]. Alkali pretreatment can improve the enzymatic hydrolysis efficiency by effectively removing lignin and increasing the fiber swelling rate [5].

Xin et al. reported that the cellulose and xylan content were increased significantly from 36.1% and 16.6% to 52.3% and 22.5%, respectively, after alkaline pretreatment [6]. The two stage of sodium hydroxide pre-extraction and then autohydrolysis pretreatment can make more than 82% of the initial sugar in the bamboo raw material covert to bioethanol through independent hydrolysis and fermentation [7].

Zhang et al. found that increasing the H_2_O_2_ loading during pretreatment led to the enhancement of substrate digestibility, whereas the alkali (only NaOH)-pretreated solid generated a higher glucose yield than that pretreated with autohydrolysis with a lower loading of H_2_O_2_ [8]. The different reagents that were used to extract lignocellulose can significantly change its physical and chemical properties, for instance reducing the lignin content in the cell wall, changing the functional group of lignin, increasing or reducing the hydrophobicity, and changing the crystallinity of cellulose [9].

Huang et al. showed that, after extraction with phosphoric acid, urea, and ethanol at room temperature, the enzymatic hydrolysis of acid-pretreated bamboo residues can be increased from 15.4% to 61.4%, 59.7%, and 42.8%, respectively [10]. Wang et al. showed that both hydrothermal pretreatment and Fenton pretreatment could effectively improve the enzymatic hydrolysis efficiency of lignocellulose and that the maximum glucose conversion rate could reach 92.4% [11].

Fenton pretreatment is an effective pretreatment method to improve the efficiency of enzymatic hydrolysis. The Fenton reaction can generate hydroxyl radicals with high oxygen intensity and lead to the reduction in the syringyl to guaiacyl ratio and phenolic hydroxyl in lignin. Fenton pretreatment increased the carboxyl group and the negative zeta potential of lignin, and reduced the non-productive adsorption of cellulase to lignin [12].

Lignin is considered as a major factor to limit the enzymatic hydrolysis of lignocellulose. Lignin physically prevents enzymes from accessing cellulose, and irreversibly adsorbs cellulase to weaken its activity, thereby, reducing the enzymatic hydrolysis [13,14,15]. The enzymatic hydrolysis of the lignocellulosic biomass begins with the adsorption of the enzyme on the fiber surface. The morphology of the lignocellulosic substrate has a major effect on the initial hydrolysis rate.

A previous study indicated that lignin redistributed and migrated to the surface of the lignocellulosic substrate during pretreatment [16]. Ju et al. proved that, compared to the total lignin content of the substrate, the surface lignin content had a direct impact on the enzymatic hydrolysis efficiency of lignocellulose [17]. Therefore, we explore the effect of the surface lignin content on the enzymatic hydrolysis of lignocellulose regarding the possible benefits to improving the efficiency of enzymatic hydrolysis.

The raw material was pretreated by different chemical methods to destroy the cell wall of lignocellulose and then to facilitate the mechanical treatment, the pretreated sample was made into high-yield pulp with a disc mill. High-yield pulping is mainly divided into mechanical pulping, chemical mechanical pulping, and semi-chemical pulping according to different mechanical treatments. At present, the bamboo high-yield chemo-mechanical pulp mainly includes hydrogen peroxide mechanical pulp (APMP), sulfonated chemical mechanical pulp (SCMP), and thermomechanical pulp (CTMP).

Compared to the bamboo raw materials, the high-yield pulp has greater advantages in producing fermentable reducing sugar, because in the high-yield pulp fiber after mechanical grinding, the cell wall structure is loose and reduced in physical obstacles, which is more conducive to the entry of enzymes. There is a lack of research on using high-yield bamboo pulp as a raw material for enzymatic hydrolysis.

Therefore, in this study, APMP, SCMP, and CTMP were the raw materials used to investigate the effects of hydrogen peroxide, Fenton pretreatment, and ethanol pretreatment on the enzymatic hydrolysis of high-yield bamboo mechanical pulp (HBMP). The surface lignin distribution and contents of different HBMPs were determined using CLSM and XPS, the correlation between the surface lignin content and enzymatic hydrolysis of HBMP was also investigated. The residue of enzymatic hydrolysis was used to adsorb methylene blue. This study provides fundamental knowledge for the enzymatic hydrolysis of high-yield bamboo pulp.

## 2. Materials and Methods

### 2.1. Materials

*Bambusa chungii* was provided by the Guangxi Academy of Forestry (Guangxi, China). The bamboo was cut into slices with a length of 2–4 cm and a width of 1–2 cm. The general chemicals used in the experiment were purchased from Tianjin Zhiyuan Chemical Pharmaceutical Co., Ltd. (Tianjin, China). All standard reagents were purchased from Sigma-Aldrich (Shanghai, China). Cellulase came from Novozymes, (Cellic CTec II, Novo Nordisk A/S, Gentofte Municipality, Demark), with an enzymatic activity of 78 FPU/g.

### 2.2. Preparation of High-Yield Bamboo Pulp

The three different chemo-mechanical bamboo pulps used include hydrogen peroxide mechanical pulp (APMP), sulfonated chemical mechanical pulp (SCMP), and chemical thermomechanical pulp (CTMP).

The preparation of APMP was as follows. The bamboo slices were soaked in water for 24 h and steamed in a digester (KRK, 2615-4, Tokyo, Japan) at 100 °C for 30 min, then the sample was processed in screw extrusion by Wenrui machinery (JS10, Anqiu, China) and then the pulp was impregnated using H_2_O_2_ treatment. The distances between the grinding discs were 0.2, 0.15, and 0.15 mm, respectively. The slurry concentration was 15%. After acidification, latency removal, and pulp screening, the required APMP was obtained [18,19].

The preparation of SCMP was as follows. The bamboo slices were soaked in water for 24 h, cooked with 15% Na_2_SO_3_ and 2% NaOH at 130 °C for 2 h, the solid–liquid ratio was 1:5. The cooked sample was refined, the distances between the grinding discs were 0.2, 0.15, and 0.15 mm, respectively, and the slurry concentration was 15% and then the slurry was washed by water to obtain the SCMP [20].

The preparation of CTMP was as follows. The bamboo slices were pretreated with hot water using a six-container rotary cooker (Greenwood Instruments, LLC., Andover, MA, USA). Each jar was filled with 40 g of the sample, the pretreatment was conducted at 130 °C for 90 min with a solid–liquid ratio of 1:15. Then, the heated samples were refined using a high-consistency refiner, the distances between the grinding discs were 0.2, 0.15, and 0.15 mm, respectively. The slurry concentration was 15%. Then, the slurry was washed by water to obtain the CTMP.

### 2.3. Pretreatments

#### 2.3.1. Hydrogen Peroxide Pretreatment

The three kinds of high-yield bamboo chemo-mechanical pulp (HBMP) were pretreated by the hydrogen peroxide pretreatment, the concentration of pulp was 10%, the pH value was 3, and 0.3% (mass fraction) ethylene diamine tetra-acetic acid (EDTA) was added at 60 °C for 1 h. Then, the chelated pulp was pretreated by hydrogen peroxide, the pulp concentration was 20%, the 6% H_2_O_2_ and 3% NaOH were added at 80 °C for 3 h, and then the pulp was washed with water to neutral for further use. The hydrogen peroxide-pretreated pulp was nominated as H-APMP, H-SCMP and H-CTMP, respectively.

#### 2.3.2. Fenton Pretreatment

The three kinds of HBMP were pretreated with the Fenton reagent. We added 16 mM FeSO_4_ (5 mL g^−1^) and 30% H_2_O_2_ (5 mL g^−1^) at pH = 3 in HBMP, the ratio of solid to liquid was 1:15, and the reaction was conducted at 25 °C for 2 h in a shaker. After the reaction was completed, the pulp was washed with water to neutral for further use. The Fenton-pretreated pulp was nominated as F-APMP, F-SCMP and F-CTMP, respectively.

#### 2.3.3. Ethanol Pretreatment

We added 10 g of HMBP into the reactor, 100 mL of 50% ethanol was added, and the reactor was placed into an oil bath at 140 °C for 1 h. After the reaction was completed, the reactor was cooled to room temperature, and the pretreated sample was washed to neutral for further use. The ethanol-pretreated pulp was nominated as E-APMP, E-SCMP and E-CTMP, respectively.

### 2.4. Chemical Composition Analysis

The chemical composition of the sample was analyzed according to the method of the American Renewable Energy Center [21]. The sample was 0.5 g, 3 mL of (72%, *w*/*w*) concentrated sulfuric acid was added, and the sample was carbonized in a water bath at 30 °C for 1 h. Then, 84 mL of distilled water was added, and hydrolyzed in an autoclave at 121 °C for 45 min. After the completion of the reaction, the liquor was filtered to obtain the solid as the acid-insoluble lignin. The liquor was measured by the ultraviolet absorbance measurement (Second plus 50, Analytik jena, Jena, Germany) at 205 nm to calculate the acid-soluble lignin. high performance liquid chromatography (HPLC) (Agilent, 1260, Santa Clara, CA, USA) was used to determine the monosaccharides in the liquor. We placed 2 g of the sample in a crucible and burned in a muffle furnace at 575 ± 25 °C for 6 h, and the ash was retained.

### 2.5. Characterization of Physicochemical Properties

Scanning electron microscopy (Phenom Pro, Eindhoven, The Netherlands) was used to observe the surface morphology of the HBMP fibers with a scanning voltage of 10 kV and a scanning magnification of 3000.

The crystallinity of the sample was analyzed using X-ray diffraction (SMARTLAB3KW, Rigaku Corporation, Japan). The detection parameters were as follows: the voltage was 40 kV and the scanning speed and frequency were 0.12 s/step and 0.02°, respectively. The scanning range was 10–50° (2θ). The crystallinity of the samples was calculated according to the Segal formula [22].

*CrI* = (I_002_ − I_am_)/I_002_ (1)

where I_002_ is the maximum intensity of the diffraction absorption peak at 2θ = 22.5°, and I_am_ is the intensity of the non-crystalline absorption peak at 2θ = 18°.

The change of functional groups in the HBMP fiber was analyzed using Fourier transform infrared spectroscopy (FTIR, TENSOR II, BRUKER, Ettlingen, Germany). We uniformly mixed and pressed into tablets 1 mg of the sample and 100 mg of potassium bromide. Then, we tested the tablets in infrared. The scanning range was 4000–400 cm^−1^, the scanning speed was 4 cm^−1^.

The fluorescence characteristics of the fiber surface was determined by the confocal laser scanning microscope (CLSM, Zeiss company LSM880, Oberkochen, Germany). We fixed the sample on the glass slide, 0.1 mL of distilled water was dripped on the sample, and then we covered the slide with a coverslip, dried the sample at room temperature, and then placed the sample on the CLSM sample stage for observation. The CLSM objective lens was 40X, the excitation wavelength was 488 nm, and the collection range of the emission spectrum was 500–550 nm (green), 550–600 nm (yellow), 600–650 nm (red), respectively.

An X-photoelectron spectrometer (XPS, ESCALAB 250Xl^+^, Thermo Fisher Scientific Corporation, Bartlesville, OK, USA) was used to determine the surface lignin content of the untreated pulp and the Fenton-pretreated HBMP. The X-ray excitation source was monochromatic A1 K Alpha (*hv* = 1486.6 eV), and the beam spot was 650 μm. The scan mode was CAS with a full spectrum scan of 100 eV and narrow spectrum scan of 30 eV, and each sample was detected at three points. The surface lignin content (SLC) was calculated according to the following formula.

SLC (%) = (O/C_sample_ − O/C_carbohydrate_)/(O/C_lignin_ − O/C_carbohydrate_) × 100% (2)

Here, O/C_carbohydrate_ is 0.83, and O/C_lignin_ is 0.33.

### 2.6. Enzymatic Hydrolysis

We added 1 g of the sample into a 50 mL conical flask, then 20 mL of acetic acid–sodium acetate buffer solution (pH = 4.8) was added; the enzyme dosage was 20 FPU/g, the enzymatic hydrolysis was conducted at 50 °C for 48 h at 150 rpm, and 0.5 mL of the enzymatic hydrolysate was used for the sugar analysis. The sampling times were 6, 12, 24, and 48 h. After the completion of enzymatic hydrolysis, the washed residue was vacuum dried and then used for the adsorption experiment of methylene blue.

### 2.7. HPLC

The sugar content in the enzymatic hydrolysate was determined using high-performance liquid chromatography (HPLC, Agilent 1260, USA) equipped with refractive index detectors (RID, 1260 Infinity II, Agilent, USA). The chromatographic column was an HPX-87P, and the mobile phase and column temperature were 5 mM H_2_SO_4_ and 80 °C, respectively. The flow rate and injection volume were 0.6 mL min^−1^ and 10 µL, respectively. To ensure the accuracy of the test, all samples were diluted with ultrapure water before testing. The standard curves of glucose and xylose were measured. The enzymatic hydrolysis efficiency (EHE) was calculated according to the following formula:

EHE (%) = (glucose + xylose) in enzymatic hydrolysate/(glucose + xylose)(3)

### 2.8. Methylene Blue Adsorption

We added 0.1 g of enzymatic hydrolysis residue into a 50 mL conical flask with 10 mL methylene blue (MB) solution (400 mg L^−1^). The adsorption experiment was conducted on a gas bath constant-temperature oscillator (CHA-S, Guohua, China) at 50 °C for 100 min with 150 rpm. After the adsorption was completed, the mixed liquor was centrifuged at 10,000 rpm for 20 min to obtain the liquor and solid, and the liquor was measured by UV-Vis spectroscopy (Specord plus 50, Analytik jena, Germany) at 663 nm. The standard curve of methylene blue was measured. The adsorbed MB content was calculated according to the following formula.

*Q_e_* = (*C_0_*−*C_e_*) *V*/*W* (4)

Here, *C*_0_ is the initial concentration of MB (g L^−1^), *C_e_* is the equilibrium concentration of MB (g L^−1^), V is the volume of the solution (L), and *W* is the mass of the enzymatic hydrolysis residue (g).

## 3. Results and Discussion

### 3.1. Morphology of Different Fibers

**Figure 1:**
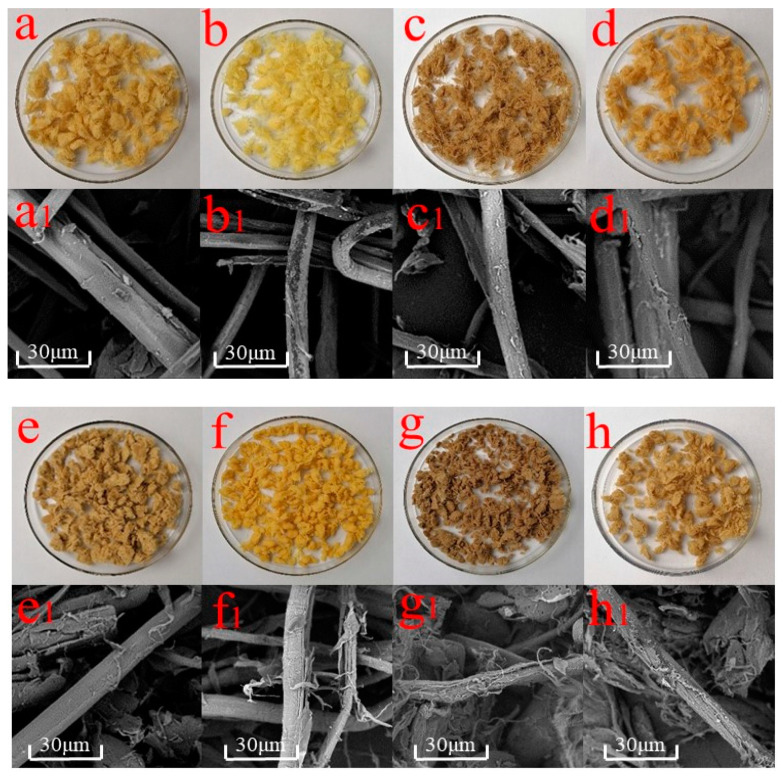

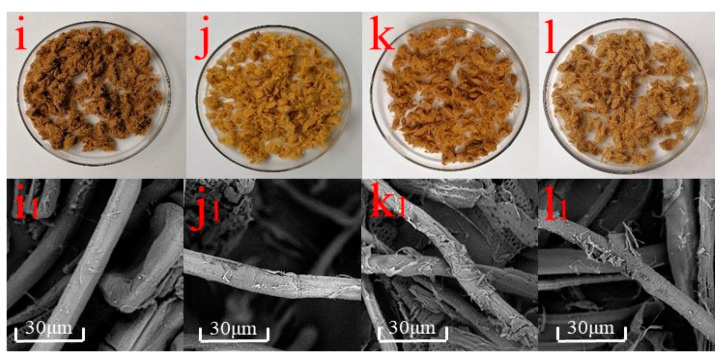
Photographs/SEM images (×2000) of samples: (**a**,**a1**) hydrogen peroxide mechanical pulp (APMP) APMP; (**b**,**b1**) H-APMP; (**c**,**c1**) F-APMP; (**d**,**d1**) E-APMP; (**e**,**e1**) sulfonated chemical mechanical pulp (SCMP); (**f**,**f1**) H-SCMP; (**g**,**g1**) F-SCMP; (**h**,**h1**) E-SCMP; (**i**,**i1**) chemical thermomechanical pulp (CTMP); (**j**,**j1**) H-CTMP; (**k**,**k1**) F-CTMP; and (**l**,**l1**) E-CTMP.

The effects of the pretreatments on the optical properties of high-yield bamboo mechanical pulp (HBMP) fibers are shown in Figure 1. CTMP fibers were deeper in color than the APMP and SCMP fibers, which may be related to more lignin on the surface of CTMP fibers. After hydrogen peroxide and ethanol pretreatments, the color of the APMP, SCMP, and CTMP fibers became lighter, while the color of the APMP, SCMP, and CTMP fibers became deeper after Fenton pretreatment, possibly due to the highly active hydroxyl free in the Fenton reagent-oxidized lignin to generate the additional carbonyl structure [23], and to form more chromophoric groups, and, therefore, the fiber color became deeper [24].

The ferric ion was adsorbed on the surface of fibers, which also resulted in the color of the fibers becoming deeper. SEM analysis showed that the surface of the HBMP fibers was smooth and flat. There were obvious flocculent fine fibers on the surface of the SCMP fibers, indicating that the damage degree of SCMP was higher than that of APMP and CTMP. After Fenton pretreatment, the cracks and the fine fibers of APMP, SCMP, and CTMP increased, compared to the hydrogen peroxide and ethanol pretreatment due to the dehydration and cleavage of cellulose glycosidic bonds after the Fenton oxidation reaction [25].

### 3.2. Chemical Compositions of Samples

The effect of pretreatment on the chemical composition of HBMP was shown in Table 1. The contents of lignin and hemicellulose in APMP were lower than in SCMP and CTMP, due to the degradation and removal of hemicellulose and lignin after hydrogen peroxide were greater than sulfonation and heat treatment. After pretreatment with hydrogen peroxide, Fenton, and ethanol, the cellulose, hemicellulose, and lignin contents of HBMP were reduced. The content of cellulose, hemicellulose, and lignin in Fenton-pretreated HBMP were lower than in hydrogen-peroxide- and ethanol-pretreated HBMP.

For example, the removal rates of hemicellulose and lignin in F-APMP were 30.2% and 33.1%, respectively. The removal rates of hemicellulose and lignin in F-SCMP were 55.3% and 23.2%, respectively. The removal rates of hemicellulose and lignin in F-CTMP were 22.3% and 25.1%, respectively. These results show that APMP removed more lignin with the Fenton pretreatment than did SCMP and CTMP, possibly due to the residual lignin in APMP that was oxidized by the Fenton reagent.

The removal of hemicellulose in SCMP by Fenton pretreatment was higher than APMP and CTMP, possibly because the surface of SCMP was damaged more by Fenton pretreatment compared with APMP and CTMP, which was conducive to the penetration of the pretreatment liquor. Previous reports showed that the hemicellulose and lignin affected the enzymatic hydrolysis efficiency of lignocellulose [26,27].

### 3.3. XRD Analysis

**Figure 2:**
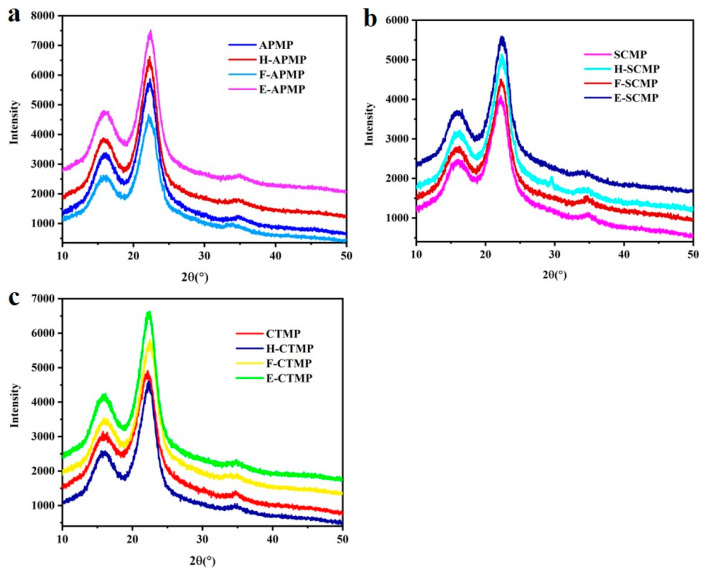
XRD analysis of the samples: (**a**) APMP samples, (**b**) SCMP samples, and (**c**) CTMP samples.

The effects of pretreatment on the crystallinity of the high-yield bamboo mechanical pulp (HBMP) are shown in Figure 2, and the crystallinity was calculated as shown in Table 2. SCMP demonstrated lower crystallinity compared with APMP and CTMP. The crystallinity of APMP, SCMP, and CTMP increased after different pretreatments, as the hemicellulose and lignin of the non-crystalline region were severely removed by different pretreatments.

For examples, the crystallinity of SCMP, H-SCMP, F-SCMP, and E-SCMP were 53.2, 65.3, 61.4, and 63.7, respectively, indicating that the crystallinity of F-SCMP was lower than that of H-SCMP and E-SCMP. This was because the degradation of cellulose in the crystalline region from the Fenton pretreatments was stronger than with the hydrogen peroxide and ethanol pretreatments, resulting in the decrease of crystallinity in the HBMP fibers. The similar changes of crystallinity were found in APMP and CTMP.

The crystallinity of F-SCMP (61.4) was higher than F-APMP and F-CTMP, as the cellulose, hemicellulose, and lignin of F-SCMP were lower than with F-APMP (59.1) and F-CTMP (58.6). Wang et al. proved that the crystallinity of lignocellulose increased after pretreatment with the Fenton reaction [28]. The enzymatic hydrolysis efficiency of lignocellulose was affected by the crystallinity [29,30].

### 3.4. FTIR Analysis

**Figure 3:**
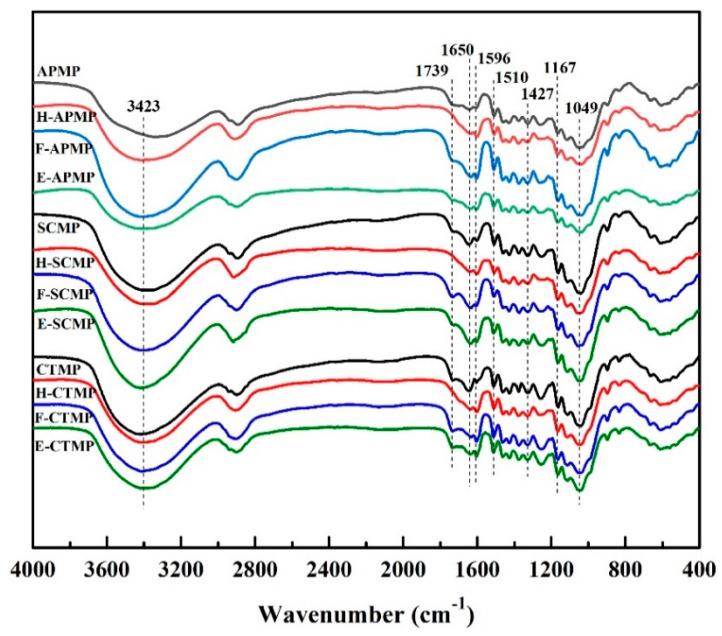
FTIR analysis of the HBMP samples.

The FTIR analysis of the different high-yield bamboo mechanical pulps (HBMP) is shown in Figure 3. The FTIR peak intensity was calibrated using the peak at 1110 cm^−1^ as a reference peak. The stretching vibration peaks at 3423 cm^−1^ were assigned to the hydroxyl and phenolic hydroxyl groups [31,32], and the stretching vibration peaks at 1739 cm^−1^ were assigned to non-conjugated carbonyl groups [33,34]. The adsorption peak at 1596, 1510, 1462, and 1427 cm^−1^ were assigned to the CH plane bending vibration of skeleton structure of aromatic ring in lignin [35,36].

Compared to the unpretreated HBMP, the functional groups in pretreated HBMP changed after different pretreatments. For example, the intensity of the hydroxyl group in SCMP was enhanced after hydrogen peroxide and Fenton pretreatment; however, the intensity of the hydroxyl group was weakened after the ethanol pretreatment, possibly due to the hydroxyl group being oxidized by the hydroxyl radical to produce a carboxyl group during the hydrogen peroxide and Fenton pretreatment. The intensity of the non-conjugated carbonyl group in H-SCMP was lower than in F-SCMP and E-SCMP, indicating that the non-conjugated carbonyl group was significantly decreased after the hydrogen peroxide pretreatment.

Compared to SCMP, the peak intensity of the skeleton structure of the aromatic ring decreased in H-SCMP and F-SCMP, but changed little in E-SCMP, indicating that hydrogen peroxide and Fenton pretreatment can effectively remove lignin from HBMP [37], the removal of lignin in HBMP by ethanol pretreatment was less. The absorption peak at 1049 cm^−1^ was assigned to the pyranose, which was decreased after the hydrogen peroxide, Fenton, and ethanol pretreatments, which implied the degradation of the carbohydrates in the fibers. These results were consistent with the component analysis of HBMP (Table 2).

### 3.5. Enzymatic Hydrolysis

**Figure 4:**
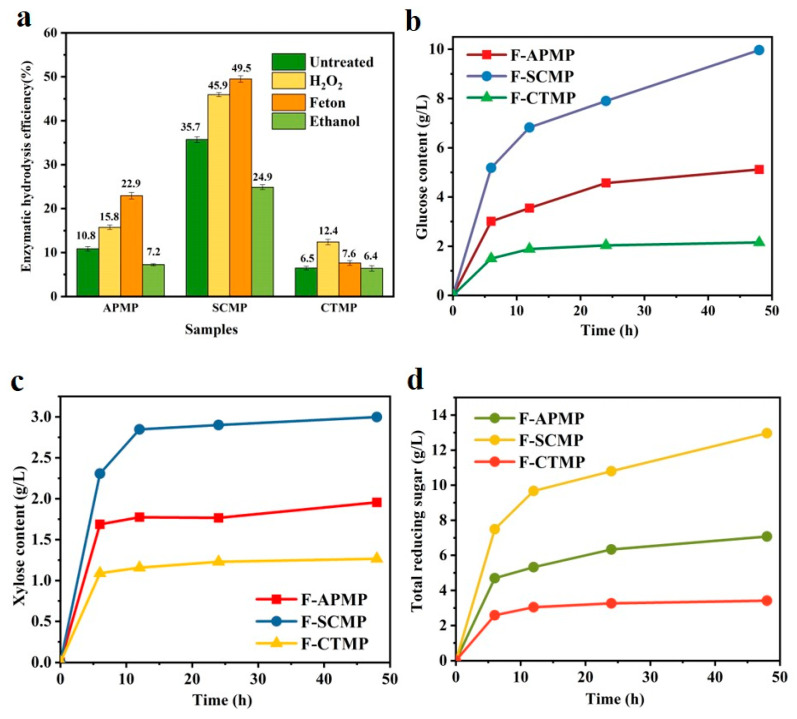
The enzymatic hydrolysis efficiency and sugar analysis of HBMP ((**a**), enzymatic hydrolysis efficiency; (**b**), glucose; (**c**), xylose; and (**d**), total reducing sugar).

The enzymatic hydrolysis efficiency of the HBMP is shown in Figure 4a. The enzymatic hydrolysis efficiency of the APMP, SCMP, and CTMP increased after the hydrogen peroxide and Fenton pretreatment; however, the enzymatic hydrolysis efficiency of the APMP, SCMP, and CTMP decreased after the ethanol pretreatment, possibly due to the removal of lignin and hemicellulose after the ethanol pretreatment. The small lignin molecule in the ethanol pretreated hydrolysate was re-adsorbed on the surface of the fiber, which increased the ineffective adsorption of lignin to cellulase and reduced the enzymatic hydrolysis efficiency.

The enzymatic hydrolysis efficiency of SCMP was higher than for APMP and CTMP, and the highest enzymatic hydrolysis efficiency of F-SCMP was 49.5%, due to the lignin and hemicellulose being removed. This led to the non-productive adsorption of lignin to cellulase being reduced and the weakened steric hindrance of hemicellulose to cellulase [38].

The SO_3_^2−^ attached to the lignin increased the hydrophilicity of lignin, decreased the hydrophobic interaction between lignin and cellulase, and may also increase the enzymatic hydrolysis. The glucose and xylose content in the enzymatic hydrolysate of HBMP are shown in Figure 4b,c, the glucose and xylose content of HBMP increased with the enzymatic time, and the glucose and xylose contents of F-SCMP were higher than for F-APMP and F-CTMP. The increased glucose and xylose content of HBMP resulted in the increase of the total reducing sugar (Figure 4d).

### 3.6. CLSM

**Figure 5:**
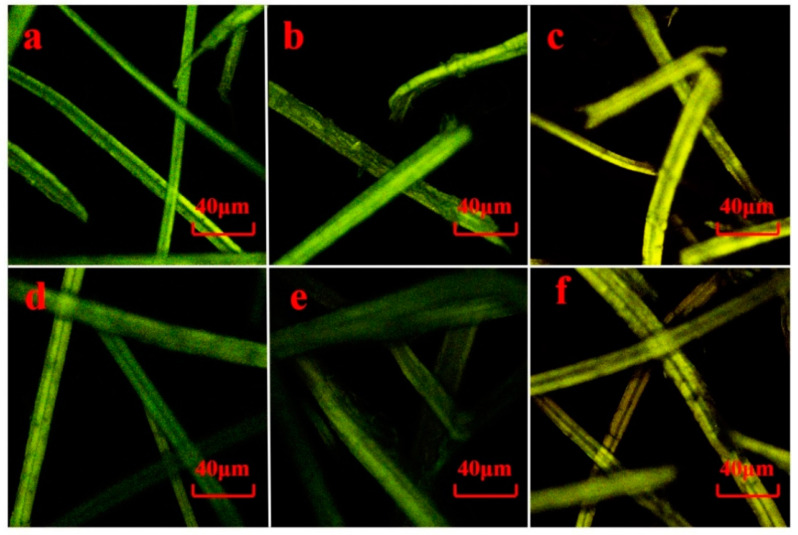
CLSM analysis of the Fenton-pretreated samples: (**a**) APMP; (**b**) SCMP; (**c**) SCMP; (**d**) F-APMP; (**e**) F-SCMP; and (**f**) F-CTMP.

Lignin as a kind of self-fluorescence polymer can be analyzed by confocal Laser Scanning Microscopy (CLSM) [39], and the effects of pretreatment on the lignin distribution on the surface of fibers are shown in Figure 5. The distribution of lignin on the surface of the fibers was nonuniform. The fluorescence intensity on the surface of the APMP, SCMP, and CTMP fibers implied the content of the surface lignin.

The fluorescence intensity on the surface of the CTMP fibers was higher than the APMP and SCMP, indicating the high lignin distributed on the surface of the CTMP fiber. Compared to the untreated APMP, SCMP, and CTMP fibers, the fluorescence intensity on the surface of the F-APMP, F-SCMP, and F-CTMP fibers was obviously weakened, indicated that the Fenton pretreatment effectively removed the lignin on the surface of the fibers and changed the distribution of the lignin. This result was consistent with the result of the component analysis (Table 2).

### 3.7. XPS Analysis

XPS was used to analyze the surface lignin content (SLC) of unpretreated and Fenton-pretreated samples, the elemental analysis is shown in Table 3. The content of C on the surface of the APMP fibers was lower than for SCMP and CTMP, and the content of O and the O/C ratio of the APMP fibers were higher than those of SCMP and CTMP. The S element content on the surface of the SCMP fibers was higher than that of APMP and CTMP, due to the initial sulfonation reaction between Na_2_SO_3_ with the fibers during the pulping process, which led to the SO_3_^2−^ attaching on the surface of the fibers, resulting in the higher sulfur content on the surface of the fibers [40].

The SLC of the CTMP fibers was higher than that of APMP and SCMP, possibly due to the large amount of lignin in the CTMP, which migrated from the interior to the surface of the fiber during the pulping process [41]. Compared with untreated HBMP, the content of the C and S elements on the surface of the F-APMP, F-SCMP, and F-CTMP fibers decreased, and the content of O increased, and the SLC of F-APMP, F-SCMP, and F-CTMP decreased by 24.93%, 15.70%, and 12.87%, respectively. A previous report indicated that the high SLC could affect the enzymatic hydrolysis efficiency of the fibers [42].

There was a linear relationship between the enzymatic hydrolysis efficiency and the SLC of APMP and CTMP. However, the high SLC decreased the enzymatic hydrolysis of HBMP. For the SCMP and F-SCMP, although the SLC values of SCMP and F-SCMP were higher than the SLC values of APMP and F-APMP, the SCMP and F-SCMP had higher enzymatic hydrolysis efficiency. This result is possibly due to the hydrophilic group of sulfonic groups that was introduced during the pulping process of SCMP, which increased the hydrophilicity of lignin, and ultimately decreased the non-productive adsorption of lignin to cellulase.

**Figure 6:**
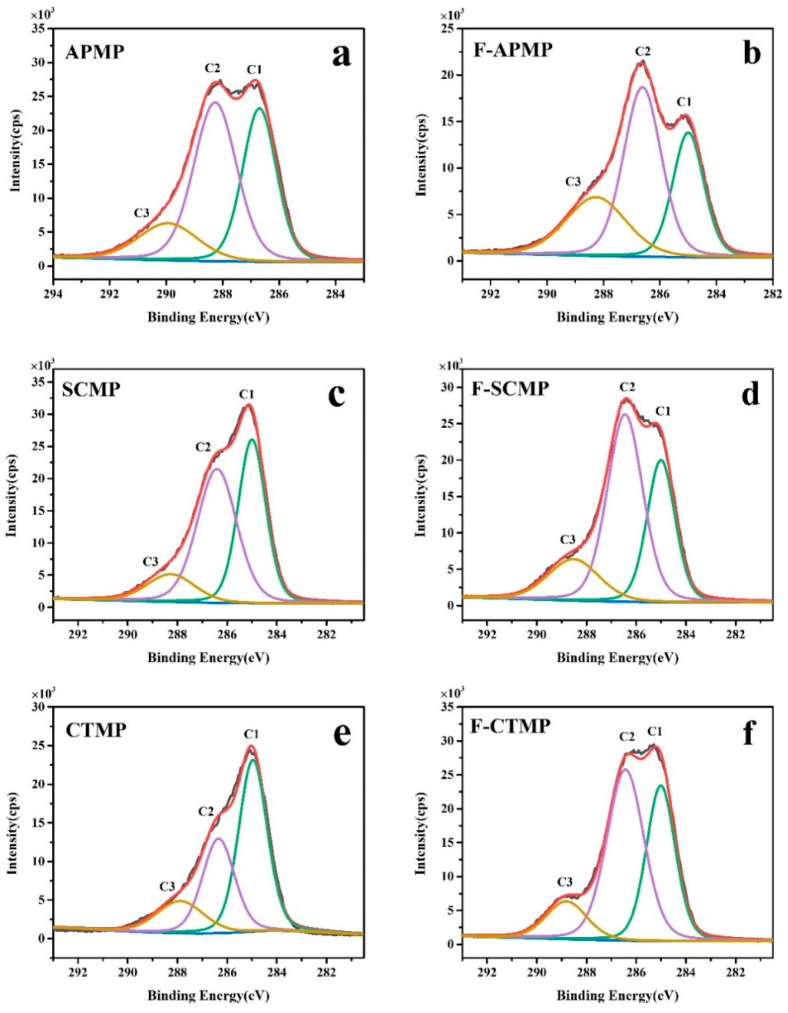
XPS analysis of the C1s peak of the pulp: (**a**) APMP; (**b**) F-APMP; (**c**) SCMP; (**d**) F-SCMP; (**e**) CTMP; and (**f**) F-CTMP.

The change of SLC was confirmed by the analysis of the C1s peak, as shown in Figure 6. The composition of C1s is shown in Table 4. The C1 was mainly derived from the lignin and extracts [43]. Compared to APMP, SCMP, and CTMP, the proportion of the C1 peak of F-APMP, F-SCMP, and F-CTMP decreased by 45.50%, 23.21%, and 0.77%, respectively, which was consistent with the change of SLC.

### 3.8. Adsorption of Methylene Blue by Enzymatic Hydrolysis Residue

**Figure 7:**
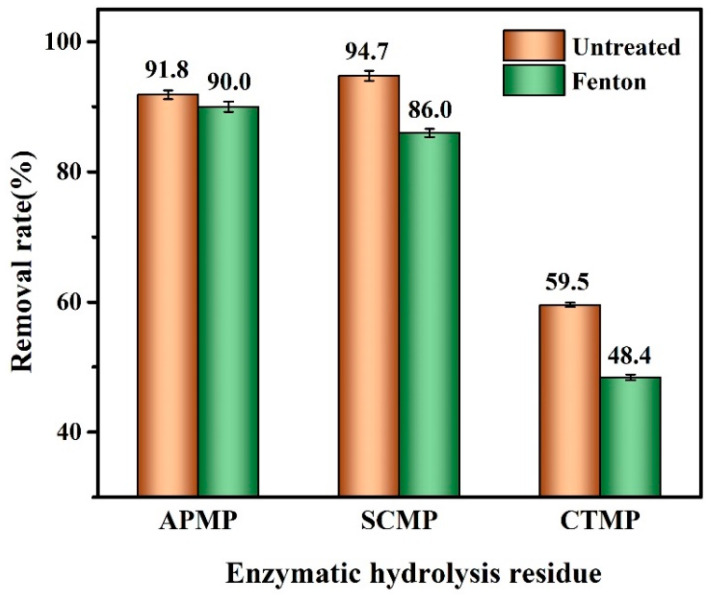
The removal rate of methylene blue by the enzymatic hydrolysis residue.

To effectively use the residual solids after enzymatic hydrolysis, the methylene blue (MB) was adsorbed by the enzymatic hydrolysis residue (EHR), as shown in Figure 7. The MB removal rates of the APMP, SCMP, and CTMP EHR were 91.8%, 94.7%, and 59.5%, respectively. The MB removal rates of Fenton-pretreated APMP, SCMP, and CTMP EHR were 90.0%, 86.0%, and 48.4%, respectively.

The EHR of SCMP adsorbed more MB when compared with APMP and CTMP, possibly due to the enzymatic hydrolysis, which removed a part of the carbohydrates from the fiber, formed more holes, and increased the specific surface area of the fiber, and thus increased the adsorption of methylene blue [44]. The surface lignin content of APMP, SCMP, and CTMP were decreased after the Fenton pretreatment, which possibly decreased the MB removal rate of the Fenton-pretreated APMP, SCMP, and CTMP EHR. This result implied that not only did the Fenton-pretreated SCMP present the highest enzymatic hydrolysis efficiency but also that the enzymatic hydrolysis residue of SCMP had a high adsorption capacity of MB.

## 4. Conclusions

Pretreatment can improve the enzymatic hydrolysis of high-yield bamboo mechanical pulp (HBMP). The enzymatic hydrolysis efficiency of HBMP was improved by Fenton pretreatment, which was higher than with the hydrogen peroxide and ethanol pretreatments. The surface lignin content of the APMP and CTMP significantly affected the enzymatic hydrolysis; however, the enzymatic hydrolysis of SCMP was simultaneously impacted by the surface lignin content and hemicellulose content.

The introduction of sulfur possibly increased the hydrophilicity of the fiber, which is beneficial in improving the enzymatic hydrolysis efficiency of the fiber. The enzymatic hydrolysis residue of HBMP had an excellent adsorption capacity of methylene blue. This work provides fundamental knowledge regarding the enzymatic hydrolysis of high-yield bamboo pulp and presents an effective method for efficient utilization of the enzymatic hydrolysis residue.

## Figures and Tables

**Table 1:** Chemical compositions of samples.

Samples	Glucose (%)	Xylose (%)	Lignin (%) ^a^
APMP	35.0 ± 0.02	11.9 ± 0.04	23.9 ± 0.05
H-APMP	33.6 ± 0.05	9.9 ± 0.01	16.3 ± 0.01
F-APMP	30.8 ± 0.04	8.3 ± 0.08	15.9 ± 0.05
E-APMP	34.9 ± 0.06	10.5 ± 0.02	20.3 ± 0.06
SCMP	34.5 ± 0.08	13.2 ± 0.02	24.6 ± 0.04
H-SCMP	30.8 ± 0.05	10.1 ± 0.04	20.3 ± 0.02
F-SCMP	28.6 ± 0.03	5.9 ± 0.04	18.9 ± 0.01
E-SCMP	34.6 ± 0.05	12.3 ± 0.02	22.4 ± 0.02
CTMP	39.2 ± 0.07	14.3 ± 0.04	25.5 ± 0.06
H-CTMP	32.9 ± 0.03	12.0 ± 0.04	21.2 ± 0.07
F-CTMP	31.3 ± 0.09	11.1 ± 0.05	19.1 ± 0.06
E-CTMP	35.5 ± 0.02	13.0 ± 0.03	24.6 ± 0.05

^a^ The lignin content (%) includes acid-soluble and acid-insoluble lignin.

**Table 2:** Crystallinity of the samples.

Sample	Crystallinity (%)
APMP	55.9
H-APMP	66.0
F-APMP	59.1
E-APMP	62.6
SCMP	53.2
H-SCMP	65.3
F-SCMP	61.4
E-SCMP	63.7
CTMP	53.7
H-CTMP	63.8
F-CTMP	58.6
E-SCMP	62.4

**Table 3:** XPS analysis of the surface elements and lignin contents of pulp.

Samples	C (%)	O (%)	N (%)	S (%)	O/C	SLC (%)
APMP	70.05	27.91	1.99	0.05	0.40	86.31
SCMP	71.03	26.85	1.47	0.65	0.34	90.40
CTMP	71.80	26.45	1.66	0.09	0.37	92.96
F-APMP	65.55	33.17	1.25	0.03	0.51	64.79
F-SCMP	68.14	30.59	1.03	0.34	0.45	76.21
F-CTMP	69.52	29.55	0.89	0.04	0.42	80.99

**Table 4:** Element composition of the C1s peak of the pulp.

Samples	C1	C2	C3
APMP	36.51	48.07	15.42
SCMP	40.63	46.47	12.90
CTMP	52.90	32.59	14.51
F-APMP	28.02	49.40	22.58
F-SCMP	31.20	52.24	16.56
F-CTMP	37.23	50.37	12.40

## Data Availability

Data available in a publicly accessible repository.
